# The mini player with diverse functions: extracellular vesicles in cell biology, disease, and therapeutics

**DOI:** 10.1007/s13238-021-00863-6

**Published:** 2021-08-10

**Authors:** Abhimanyu Thakur, Xiaoshan Ke, Ya-Wen Chen, Pedram Motallebnejad, Kui Zhang, Qizhou Lian, Huanhuan Joyce Chen

**Affiliations:** 1 The Pritzker School of Molecular Engineering, The University of Chicago, Chicago, Illinois USA; 2 The Ben May Department for Cancer Research, The University of Chicago, Chicago, Illinois USA; 3 Division of Pulmonary, Critical Care and Sleep Medicine, Department of Medicine, Keck School of Medicine, Hastings Center for Pulmonary Research, University of Southern California, Los Angeles, CA 90089 USA; 4 Department of Stem Cell Biology and Regenerative Biology, Keck School of Medicine, University of Southern California, Los Angeles, CA 90089 USA; 5 Department of Medicine, Li Ka Shing Faculty of Medicine, The University of Hong Kong, Pok Fu Lam, Hong Kong; 6 Prenatal Diagnostic Center and Cord Blood Bank, Guangzhou Women and Children’s Medical Center, Guangzhou Medical University, Guangzhou, China; 7 HKUMed Laboratory of Cellular Therapeutics, the University of Hong Kong, Pok Fu Lam, Hong Kong

**Keywords:** extracellular vesicles, exosomes, stem cells, cancer, exosomal communication, exosomal therapeutics

## Abstract

Extracellular vesicles (EVs) are tiny biological nanovesicles ranging from approximately 30–1000 nm in diameter that are released into the extracellular matrix of most cell types and in biofluids. The classification of EVs includes exosomes, microvesicles, and apoptotic bodies, dependent on various factors such as size, markers, and biogenesis pathways. The transition of EV relevance from that of being assumed as a trash bag to be a key player in critical physiological and pathological conditions has been revolutionary in many ways. EVs have been recently revealed to play a crucial role in stem cell biology and cancer progression via intercellular communication, contributing to organ development and the progression of cancer. This review focuses on the significant research progress made so far in the role of the crosstalk between EVs and stem cells and their niche, and cellular communication among different germ layers in developmental biology. In addition, it discusses the role of EVs in cancer progression and their application as therapeutic agents or drug delivery vehicles. All such discoveries have been facilitated by tremendous technological advancements in EV-associated research, especially the microfluidics systems. Their pros and cons in the context of characterization of EVs are also extensively discussed in this review. This review also deliberates the role of EVs in normal cell processes and disease conditions, and their application as a diagnostic and therapeutic tool. Finally, we propose future perspectives for EV-related research in stem cell and cancer biology.

## INTRODUCTION

Diverse communication systems mediate intercellular communication, both in physiological and pathological conditions, such as cellular junctions (tight junction, adherence junctions, gap junctions, and desmosomes), integrins, and selectins (Martin et al., 2013). Recently, a novel way of cell-cell communication mediated by extracellular vesicles (EVs) has gained lots of attention (Sverdlov 2012; Maia et al., 2018; Stahl and Raposo 2018). EVs are a heterogeneous group of cell-derived membranous nanovesicles with diameter around 30–1000 nm (sometimes as large as a few micrometers; Raposo et al., 1996). They are released from most cell types and biofluids such as blood, saliva, breast milk, semen, urine, cerebrospinal fluid (CSF), colostrum (de la Torre Gomez et al., 2018), tears (Grigoreva et al., 2017), bronchoalveolar fluid (Torregrosa Paredes et al., 2012), epididymal fluid (Gatti et al., 2005), amniotic fluid (Asea et al., 2008), bile (Masyuk et al., 2010), blastocoel fluid (Battaglia et al., 2019), middle ear effusion (Val et al., 2017), and ascites (Zhu et al., 2018). EVs can be broadly classified into exosomes, microvesicles (MVs), and apoptotic bodies (ABs), based on different biological properties such as biogenesis pathways, size and biomarker (Zhang et al., 2019).

**Figure 1:**
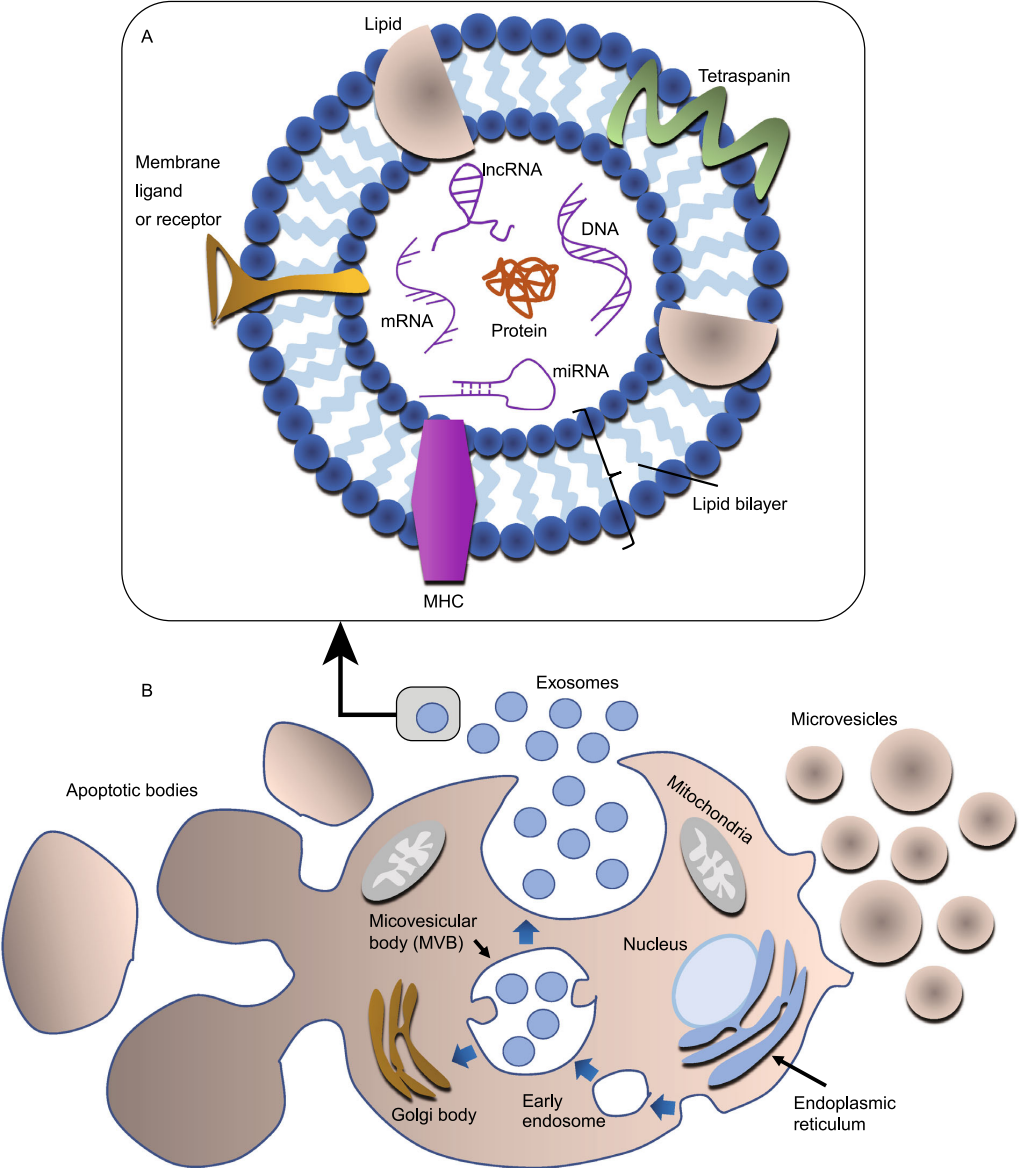
**Schematic illustration of the biogenesis of EVs and the structure of an exosome**. (A) A representative diagram of a typical exosome and its components. In general, the composition of a typical exosome differs significantly; some of the constituents can be found in a specific set of exosomes. (B) A representative diagram showing the biogenesis pathway of various EVs, including exosomes, MVs, and ABs; the release of exosomes follows an endosomal pathway; MVs are released by budding and shedding off the cell membrane, and ABs are released from the apoptotic cells

Exosomes are a subset of EVs with a size of approximately 30–100 nm, which were found to be secreted upon the fusion of multivesicular bodies (MVBs) with the plasma membrane during the differentiation of reticulocyte for the first time (Harding et al., 1984; Pan et al., 1985). Further confirmation was provided by a similar pathway of exosomes in B-lymphocytes (Raposo et al., 1996), dendritic cells (Zitvogel et al., 1998), and many other cells including platelets, T-cells, mast cells, neurons, Schwann cells, oligodendrocytes, and epithelial cells (Simons and Raposo 2009; Théry et al., 2009; Becker 2014). In addition, an *in vivo* study demonstrated the release of exosome-like vesicles from prostate epithelial cells, referred to as prostasomes and thought to be essential for motility of spermatozoa (Ronquist and Brody 1985). Common biomarkers of exosomes include tetraspanins (CD9, CD63, and CD81), heat shock proteins (HSPs), membrane transporters and fusion proteins (e.g., GTPases, annexins, and flotillin), MVB biogenesis proteins (e.g., Alix and TSG101), phospholipases, and lipid-related proteins (Conde-Vancells et al., 2008; Subra et al., 2010). Exosomes are also enriched with lipids such as cholesterols, phospholipids, biphosphates, and sphingolipids (Kao et al., 2020). Although exosomes are heterogenous (Bernard et al., 2018; Kalluri and LeBleu, 2020) and their constituents resemble their cells of origin (Salomon et al., 2015), they are also shaped by the biogenesis pathway. The biogenesis of exosomes takes place via an endosomal pathway in which the introversion of early endosomes leads to the formation of MVBs carrying intraluminal vesicles, followed by the fusion of MVBs with the plasma membrane, resulting in the excretion of exosomes in the extracellular milieu (Johnstone et al., 1987). Schematic illustrations in Fig. 1A and 1B show a typical exosomal composition and different biogenesis pathways taken by different types of EVs, respectively.

MVs are generally about 2 µm in size and produced by budding and shedding of the cell membrane via a calcium-dependent pathway. Cells undergoing apoptosis release another set of EVs with size range of 1–5 µm, referred to as ABs (Dignat-George and Boulanger 2011; Colombo et al., 2014). There is no consensus about the nomenclature of exosomes and MVs because even nanovesicles that are similar in size to exosomes and released by budding off the plasma membrane are defined as exosomes (Booth et al., 2006). Therefore, we have used the more general term “EVs” in this review.

Scientists have recently discovered a wide range of biological or physiological activities of EVs in a diversity of fields including stem cell biology, cancer biology and therapy (Tai et al., 2019; Khawar et al., 2019). This review introduces the biology and historical perspective of EVs, the role of the crosstalk between EVs produced by stem cells and stem cells-niche, and EV-mediated communication between different germ layers in developmental biology. Further, the role of EVs in cancer progression will be elaborated, followed by a discussion of the application of EVs as therapeutic agents and delivery vehicles. The role of EVs in normal cell processes and disease conditions and their application as diagnostic and therapeutic tools will also be detailed. Finally, various methods for EV characterization will be discussed in the context of technological advancements in EV-associated research.

## EVs AND THEIR CROSSTALK IN STEM CELL NICHE

Stem cells are a unique set of cells with the ability to self-renew. A single stem cell can produce clonal cell populations and further differentiate into several cell types (Tran and Damaser, 2015). Stem cells are broadly classified into embryonic stem cells (ESCs) and somatic stem cells (SSCs; also referred to as adult stem cells). ESCs are pluripotent and can differentiate into any type of cell including neural stem cells (NSCs), mesenchymal stem cells (MSCs), hematopoietic stem cells (HSCs), and endothelial progenitor cells (EPCs). SSCs were recently reported to exist in most adult tissues, maintaining tissue regeneration, turnover, and repair during injury, and are restricted to a specific lineage.

**Figure 2:**
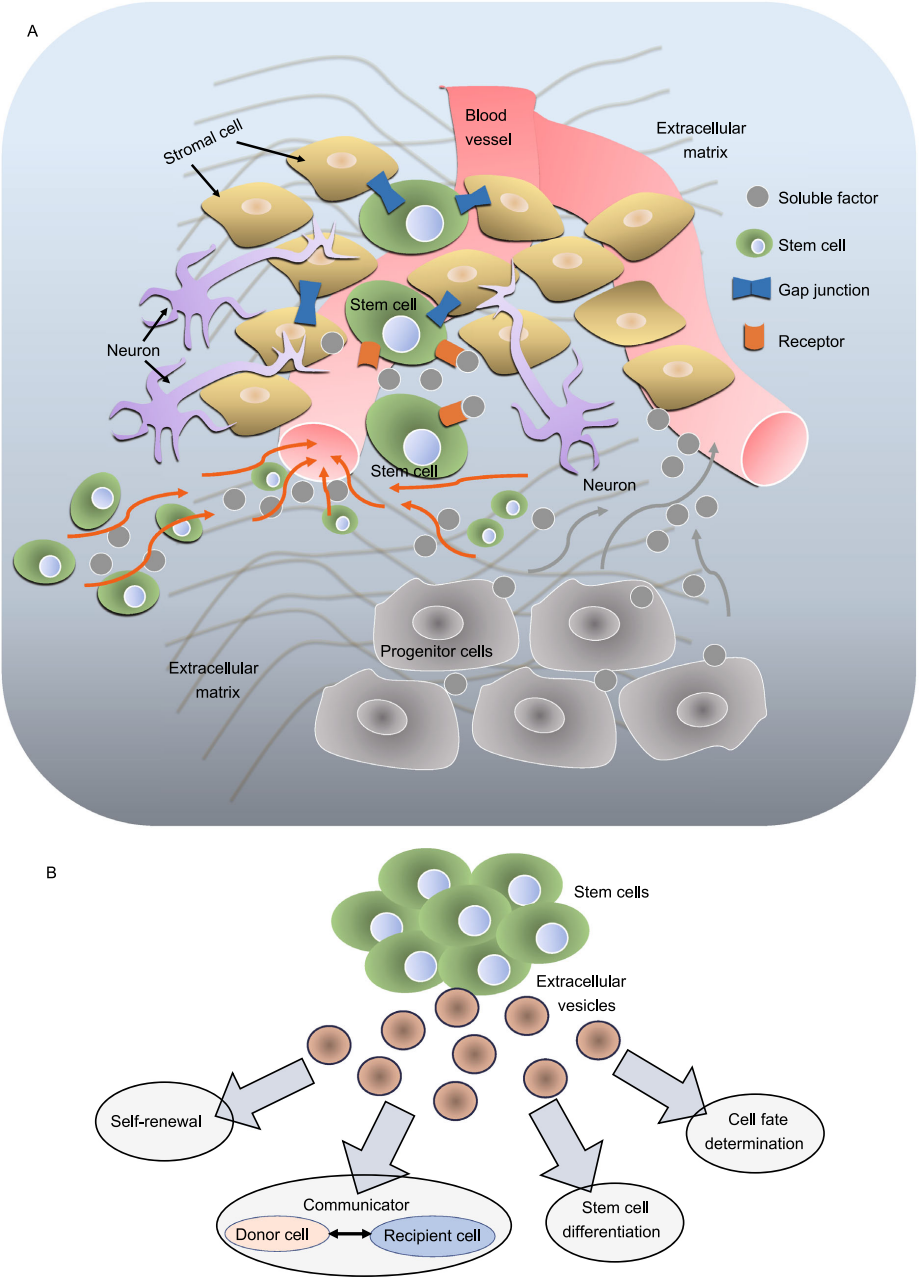
**Components of stem cell-niche and the crosstalk between stem cells and stem cell-niche via EVs**. (A) A representative diagram showing proposed stem cell-niche forming a dynamic microenvironment based on constituents reported previously. A typical stem cell-niche consists of stem cells, stromal cells, extracellular matrix, soluble factors, neural inputs, a network of blood vessels, and other components including cell adhesion molecules. Different stem cell-niches consist of several other additional components. Nonetheless the components mentioned above are necessarily present in the stem cell-niche for various functions including support and structural information. Adapted from (Jones and Wagers, 2008). (B) A representative diagram depicting various roles of EVs produced from stem cells

The fate of embryonic and adult stem cells is rigorously controlled by their microenvironment, known as stem cells-niche, through either inter- or intra- cellular communication (Pennings et al., 2018). Anatomically, stem cell niche refers to the location where the basic unit of the physiology of tissue is formed by combining signals for a balanced response from stem cells depending on the requirements of the organism (Ohlstein et al., 2004; Li and Xie, 2005). Extensive work has been carried out in various organisms to characterize the stem cell-niche (Kirkeby et al., 2016; Hsu et al., 2019). The hypothetical components of the stem cell niche include stem cells, stromal cells, extracellular matrix (ECM), and blood vessels (Pennings et al., 2018; Fig. 2A). The presence of each is not mandatory in a stem cell niche, although its structural organization and constituent elements provide a dynamic environment in which to control the cell number (stem cell pool) and the function of stem cells (Jones and Wagers, 2008). Interestingly, EVs produced from stem cells have recently attracted great attention owing to their essential roles in stem cell biology, such as regulation of self-renewal capacity (Ratajczak et al., 2006), mediating crosstalk (Deregibus et al., 2007; Camussi et al., 2010) between stem cells and niche (Hur et al., 2020), germ layer communication in organogenesis and development, and functions in stem cell differentiation and determination of cell fate (Aliotta et al., 2010; Quesenberry et al., 2010; Fig. 2B).

### Regulation of self-renewal capacity of stem cells

EVs play a crucial role in regulating the self-renewal capacity of stem cells. To maintain proper functionality as well as self-renewal capacity, the crosstalk within the stem cells-niche is of prime importance. For example, mouse embryonic stem cell EVs (mESCEVs) facilitate the sustenance of self-renewal and expansion of adult stem cells; hematopoietic progenitor cells (HPCs) via delivery of specific mRNAs (such as octamer-binding transcription factor 4 (*Oct-4*), *Nanog*, *Rex-1*, stem cell leukaemia (*SCL*), and GATA binding protein 2 (*GATA-2*)) and proteins (such as Wnt family member 3 (WNT3) and OCT4) induce the overexpression of genes responsible for pluripotency. The mESCEVs increase survival and expansion of HPCs by inducing phosphorylation of mitogen-activated protein kinases (MAPK) p42/44 and serine-threonine kinase AKT. Further, the self-renewal phenotype of HPCs diminishes after inhibition of the function of mESCEVs by heat inactivation or pre-treatment with RNase, suggesting the critical effect of the protein or mRNA cargo within the mESCEVs on maintaining stemness of HPCs (Ratajczak et al., 2006).

### Crosstalk between donor and recipient cells via EVs

The secreted factors in the stem cell-niche guide the fate of stem cells by facilitating communication, locally or remotely, by diffusing through the niche (Jones and Wagers, 2008). EVs have emerged as another way of communication (Camussi et al., 2010) between stem cells and the niche (Hur et al., 2020). Emerging evidence shows that EVs transport and deliver mRNAs that can reprogram target cells and modulate the phenotype of recipient cells (Ratajczak et al., 2006). Delivery of mRNA by EVs induces transcription in the recipient cells, and causes tissue-specific alterations (Aliotta et al., 2010), suggesting that mRNA in EVs is indeed functional. For example, EPC-derived EVs have been found to induce angiogenesis in terminally differentiated endothelial cells and proangiogenic character in dormant endothelial cells (Deregibus et al., 2007). Stem cells can also donate organelles including mitochondria (500–1000 nm) to neighbour cells to regenerate damaged tissues and cells (Zhang et al., 2016b; Jiang et al., 2019, 2016).

In addition, fibroblast-derived EVs can transport epidermal growth factor activity in the intestinal stem cell niche by carrying amphiregulin (Oszvald et al., 2019). Prostate cancer cell (PCa)-derived EVs carry cancer associated fibroblasts (CAF) that play a crucial role in communication between PCa and the tumor microenvironment (TME) to support pathways associated with stemness (Kato et al., 2017). In fact, EVs from PCa with AKT kinase activity have been reported to be taken up by normal prostate fibroblasts and induce their reprogramming via activation of stromal Myc pro-oncogene (Minciacchi et al., 2017).

### Stem cell differentiation and cell fate determination via EVs

EVs produced from stem cells also play a significant role in the differentiation of stem cells and the determination of cell fate. The constituents of EVs are capable of affecting and determining the cell fate of impending populations of recipient cells via a stable alteration in genetic makeup (Quesenberry et al., 2010; Aliotta et al., 2010). Stem cells from embryos or adult stem cells originating from diverse adult tissues, including bone marrow, liver, and adipose tissue, and induced pluripotent stem cells (iPSCs), secrete EVs (Chen et al., 2017b). Quesenberry et al., proposed that the transfer of genetic information via EVs could be a key factor in the stem cell continuum model, where the decision of stem cells to differentiate was facilitated by the transit of cell cycle and environmental stimuli (Quesenberry et al., 2010). Jung et al., showed that EVs released during the differentiation of stem cells into white or beige adipocytes could promote cell reprogramming via miRNAs (Jung et al., 2020). Stronati et al., demonstrated that EVs could induce differentiation of neural stem progenitor cells (NSPCs). The NSPCs secreted EVs during proliferation as well as after differentiation, and treatment of proliferating NSPCs with EVs derived from differentiated NSPCs induced cell differentiation in a concentrated dependent manner (Stronati et al., 2019).

### Therapeutic potential of EVs as a delivery carrier

The role of EVs as a therapeutic agent and a potential delivery vehicle has also been explored extensively. Like other EVs, stem cells EVs also recapitulate their parent cells, but are relatively less immunogenic and capable of crossing biological barriers (Bellavia et al., 2018), suggesting their therapeutic potential (Arslan et al., 2013; Ibrahim et al., 2014). Importantly, several clinical studies of EVs have been conducted, for example allogenic MSC-derived EVs were used in patients with acute ischemic stroke. ESCs-EVs carry various cargoes including miRNAs (miR-291, -294 and -295) and proteins (heat shock protein 90 (HSP90), delta like canonical Notch ligand 4 (DLL4), stress inducible protein (STI1), and Wnt-10b), and these cargoes cause cardiac regeneration, inhibition of glioblastoma growth, and retinal regeneration (Kanellopoulou et al., 2015; Khan et al., 2015; Cruz et al., 2018a; Peng et al., 2018; Wiklander et al., 2019; Zhu et al., 2019). Adipose tissue derived MSC-EVs also carry cargoes including miRNAs (miR-223, -146b, -126, -199a, -let7b, and -let7c) and proteins (CD9, CD63, HSP70, and CD81) that cause skeletal muscle regeneration, neovascularization, fibrosis inhibition, wound healing, and anti-inflammation. Umbilical cord-derived MSC-derived EVs carry miRNAs (miR-21, -146a, -181, -302a and -410) and proteins (CD63 and CD90) that cause anti-tumor and anti-apoptotic activity, neovascularization, and regulation of insulin resistance (Yao et al., 2019).

## ROLE OF EVs IN INVERTEBRATES: A MIRROR TO THE POTENTIAL ROLE IN ANIMAL MODELS

Several scientists have developed and explored the possibility of utilizing a compliant invertebrate model to study the composition of EVs (Russell et al., 2020). Compared with complex mammalian models, *Caenorhabditis elegans* (*C*. *elegans*) and *Drosophila* (Beer and Wehman, 2017) can serve as a platform for isolation of EVs actively secreted into their extracellular space. Studies in classical genetic model organisms like *C*. *elegans* and *Drosophila* have begun to reveal the developmental and behavioural roles of EVs. These organisms help decipher the signalling function of EVs in multicellular organisms. Transgenic technologies and live imaging in *C*. *elegans* and *Drosophila* facilitate the *in vivo* monitoring of EVs via reporter genes such as fluorescence or luciferase genes (Panáková et al., 2005). These *in vivo* systems can be applied to explore the potential of EV-based signalling in the developmental process, behaviour change, or disease progression in metazoans. Moreover, the molecular mechanisms that underlie the release of EVs can be studied in-depth with these models. Samuel Liégeois et al., demonstrated the role of EVs in the normal development of the *C*. *elegans* cuticle. The release of lapidated morphogens in EVs was essential to the development of the cuticle (Liégeois et al., 2006). Moreover, EVs contain lipid-modified Hh-related proteins and the transmembrane protein CHE-14/Dispatched, which is involved in Hh secretion. Interestingly, Hh-related proteins are entrapped in MVBs aggregated in the cytoplasm when the release of EVs is inhibited via depletion of membrane-bound V0 sector of the vacuolar H^+^-ATPase (V-ATPase). Cuticle development is blocked upon depletion of V-ATPase, suggesting that EV-mediated release of Hh-related proteins in *C*. *elegans* provides a unique way to transport lipid-modified or transmembrane proteins during development (Liégeois et al., 2006).

Using *Drosophila* as a model, Koles et al., demonstrated the *in vivo* mechanisms of Evenness Interrupted (Evi)-EVs release (Koles et al., 2012). In addition, Korkut et al., showed a cellular mechanism through which a secreted WNT was carried across synapses by *Wnt*-binding protein and Evi-containing vesicles, trafficking between both the WNT-producing and the WNT-receiving cells (Korkut et al., 2009). The formation of EVs was also found to be essential for developing wings in *Drosophila* via Wingless (*Wg*) and *Hh* signalling (Gross et al., 2012; Gradilla et al., 2014; Matusek et al., 2014). Further, it has been found that filopodia in *Xenopus* embryos can act as super-highways for EVs *in vivo*, indicating a potential association between cellular extensions and EVs (Danilchik et al., 2013). Overall, EVs are involved in various signalling pathways by transporting morphogens across numerous cells as demonstrated in *Drosophila*.

On the contrary, abnormal secretion of EVs can impede the development of metazoans (Wehman et al., 2011). During embryogenesis, cell division occurs followed by a change in shape and cells migrating to a suitable location in the body. Interestingly, the loss of phosphatidylethanolamine (PE) flippase TAT-5 enhances the release of EVs, leading to disruption of cell adhesion and morphogenesis of *C*. *elegans* through lipid asymmetry (Wehman et al., 2011). EVs also play a vital role in the physiology of single-cell organisms (e.g., *Chlamydomonas*), multicellular organisms (e.g., *C*. *elegans*), and mammals (Wood and Rosenbaum, 2015). For example, in *Dictyostelium*, EVs released from cilia carry transmembrane protein crucial for sexual reproduction (Cao et al., 2015). The EVs derived from ciliated neurons in *C*. *elegans* carry proteins that enable mating behaviour of the male (Wang et al., 2014a; Maguire et al., 2015). EVs also play a crucial role in the mating behaviour of *Drosophila*. EVs released from large accessory gland cells in male *Drosophila* fuse with sperm after mating and interact with female reproductive tract epithelial cells. This inhibits female remating behaviour and contributes to the reproductive advantage of the first male to mate (Corrigan et al., 2014)*.*

In the context of neuroscience, glial cells employ EVs to regulate various processes including formation of the myelin sheath and post-injury repair. EVs also facilitate the propagation of inflammatory signals induced by disease and tissue damage. They have also been reported to be associated with communication in the nervous system of intact organisms. For example, at the neuromuscular junction (NMJ) of *Drosophila*, Wnt signals are carried by EVs from neurons to muscles (Budnik et al., 2016). At the sensory neurons of *C*. *elegans,* EVs also conduct behaviourally relevant signals, for example the cilia of sensory neurons release EVs carrying the polycystin receptors, and the addition of these EVs to the rearing medium of naive animals enhances the frequency of locomotor reversal and tail chasing behaviour; a strategy employed during mating (Wang et al., 2014a; Budnik et al., 2016). Collectively, the numerous roles of EVs in classic genetic model organisms such as *C*. *elegans* and *Drosophila* may be a potential area of research to further determine their role in animal models.

## EV-MEDIATED COMMUNICATION IN EMBRYONIC DEVELOPMENT

During embryonic development, various cell types are generated to form tissues and organ systems in an organism. The inner cell mass (ICM) of the blastocyst, which produces the entire embryo (*in vivo* condition), can be isolated and cultured as ESCs with the capacity of complete development. The ICM plays a crucial role by receiving various autocrine- or paracrine-(from neighbouring cells, including trophoblast or primitive endoderm) signals to facilitate the processes that lead to embryogenesis (Plusa and Hadjantonakis, 2014). Interestingly, intercellular communication in the ICM is via EVs. Recent evidence suggests that EVs transport morphogens like Sonic Hedgehog (SHh), and miRNAs like miR-21, and causes alteration in the phenotype of recipient cells like dendritic cells (Song et al., 2018; Sun et al., 2018). EVs also facilitate communication between the trophectoderm (TE) and ICM, traversing along filopodia that spread across the blastocoel associated with mural TE to the ICM in the mouse blastocyst (Alberti and Cochella, 2017; Gross et al., 2017). Several reports have shown that the filopodia-like protrusion in the cell membrane acts as a conduit during embryogenesis (Fairchild and Barna, 2014; Sagar et al., 2015), through signalling pathways such as *Notch*, *SHh*, Decapentaplegic (*Dpp*), *EGF*, *FGF*, *Wn*t, and bone morphogenetic proteins (*BMPs*; Heckman and Plummer, 2013; McMahon and Hasso, 2013), and controls the release and delivery of EVs (Cruz et al., 2018b). Video microscopy analysis has demonstrated that EVs move along filopodia and are rearranged in the dynamic cytoskeleton via signal transduction activity by the receptors fibroblast growth factor receptor 2 (Fgfr2) and Erb-B2 receptor tyrosine kinase 3 (ErbB3; Salas-Vidal and Lomelí, 2004). Mouse ESC-secreted EVs were shown to be internalized by trophoblasts, leading to stimulation of trophoblast migration via the instigation of c-Jun N-terminal kinase (JNK) and JNK pathways. Furthermore, when injected into the blastocysts, the ESC-derived EVs enhanced the implantation ability of blastocysts in the uterus. This indicated that intercellular communication through EVs plays a crucial role to facilitate the interaction between ESCs and trophoblast during pregnancy (Desrochers et al., 2016).

### EVs in Hh signalling

The core components of the hedgehog (Hh) signalling pathway are Hh ligand, transmembrane receptor Patched 1, intracellular G-protein Smoothened and transcription factors GLI1-3. Tanaka et al. showed that FGF signalling promotes secretion of EVs containing SHh and retinoic acid at the node of the gastrula’s primitive streak. They also revealed that fluid flow at the node promoted EV movement towards the left in embryos. The asymmetric accumulation of EVs specifically activated the non-canonical Hh signalling pathway in the left of the embryo, resulting in differential changes to intracellular calcium levels and gene expression, leading to a left-right gradient of morphogens (Tanaka et al., 2005).

### EVs in Wnt signalling

Wnt signalling is involved in biological processes throughout embryonic development, such as proliferation, renewal, migration, differentiation, and polarity of stem cells (Loh et al., 2016). EVs are involved in Wnt signalling during extracellular transport in *Drosophila* through argosomes, where Wnt is released via EVs (Greco et al., 2001). Several reports indicate that EVs carry released Wingless (Wg) in neuromuscular junction and fly larval imaginal disc. The release of Wg has been found to be associated with Wg transmembrane binding protein Evi/Wntless (Wls)/Sprinter (Srt) and Wg is carried by Evi-containing EVs via synapses at the larval neuromuscular junction (KKorkut et al., 2009; oles et al., 2012). This entire process takes place during synaptic development (Koles et al., 2012).

### EVs in Notch signalling

Notch is a transmembrane protein and its signalling is involved in various key developmental processes such as cell differentiation, decision, patterning, and polarity (Mašek and Andersson, 2017). The intracellular domain of Notch is cleaved after binding of the Notch ligand to its receptor and is translocated to the nucleus, so regulating the downstream processes. The involvement of EVs in Notch signalling was speculated when Delta, one of the Notch ligands, was identified in EVs (Le Borgne and Schweisguth, 2003; Kopan and Ilagan, 2009). Delta-like 4 (DLL4) was found in EVs derived from human endothelial cells. The EVs carrying DLL4 were taken up by other endothelial cells with subsequent increased angiogenesis upon inhibition of Notch signalling. Nevertheless the underlying mechanism responsible for inhibition of Notch signalling by EVs carrying DLL4 is unknown (Sheldon et al., 2010).

### EVs in BMP signalling

Bone morphogenetic proteins (BMPs) belong to the superfamily of transforming growth factor-beta (TGF-β), responsible for morphogen-regulating organization of the body axis, tissue patterning, and development and preservation of stem cell niches at the time of embryonic development in diverse species. On the surface of the cell, BMPs bind to the receptors; bone morphogenetic protein receptors (BMRPs), by promoting the phosphorylation of members of the SMAD family that are translocated into the nucleus and regulate the expression of downstream genes (Wang et al., 2014b; Bier and De Robertis, 2015). The EVs derived from embryos of zebrafish were found to carry BMP2/4 that could activate gene expression of recipient cells. In addition, upon inhibiting the release of EVs, phosphorylation of SMAD1/5/9 and further activity of transcription were reduced, resulting in severe dorsalization phenotypes, reminiscent of disrupted BMP-signalling. This showed the crucial role of EVs in establishing the BMP morphogen gradient throughout the development of zebrafish (Draening et al., 2018).

## EVs IN NORMAL CELL PROCESSES AND DISEASE CONDITIONS

Evidence has shown that EVs are crucial for physiological and pathological functions via cell-cell communication (De Toro et al., 2015; Thakur et al., 2017). In the normal physiological system, EVs play an essential role in the immune system (Raposo et al., 1996) and in intercellular communication between various immune cells (Corrado et al., 2013). The EVs in the immune system are capable of eliciting both immune activation and immunosuppression effects. For example, EVs released from mature dendritic cells (DCs) carry major histocompatibility complex (MHC) membrane molecules, causing an adaptive immune response via activation of T cell receptors. Especially during infection, the antigens are taken up by DCs, followed by discharge of MHC complexes, and this phenotype activates helper T cells and B cells, leading to the enhanced release of EVs containing MHC complexes in B cells. In addition, the DC-derived EVs also act as a carrier for antigens between neighboring DCs. Moreover, the EVs released from B cells can stimulate CD4^+^ T cells, implying that B cell-derived EVs are also crucial to facilitate modulation of the immune response. EVs released from immature DCs are involved in suppression of the adaptive immune response by causing apoptosis of T cells and a tolerogenic immune response. By inducing differentiation of T helper cells into regulatory T cells, EVs can also mediate the balance of pro- and anti-inflammatory effector T cells (Corrado et al., 2013).

EVs have been reported to play a crucial in the central nervous system (CNS), including the brain. The intercellular communication via neuronal EVs in neurons is vital in supporting various functions of the nervous system including myelination and maintenance of axonal integrity. The release of EVs is promoted by glutamate neurotransmitter via stimulation of ionotropic glutamate receptors, followed by the uptake of these EVs that transport their constituents to neuronal cells. Further, the released EVs from oligodendrocytes can protect neurons by elevating their tolerance to stress (Frühbeis et al., 2013; Fröhlich et al., 2014).

In the cardiovascular system (CVS), cardiac muscle cells release EVs, especially under hypoxic conditions (Gupta and Knowlton, 2007). The stressed cells under hypoxic conditions release EVs containing specific constituents, including TNF-α (Yu et al., 2012), that promulgate an inflammatory response. These EVs also carry genetic material and transfer between adjacent cells, leading to alteration of gene expression in recipient cells. This explains why EV-mediated intercellular communication is not specific to a cell type in the heart (Waldenström et al., 2012; M. and Das, 2014).

EVs are crucial to sustain both physiological and pathological functions through cell-cell communication (De Toro et al., 2015; Thakur et al., 2017). As well as regulation of normal cellular processes, EVs facilitate the progression of various pathological conditions such as cancer, neurodegenerative diseases, cardiovascular diseases, and infectious diseases (De Toro et al., 2015). In order to accomplish their physiological or pathological functions, EVs must be released from one cell type (donor cell) and be taken up by another (recipient cell); depending on whether this uptake is by donor cells or by different cells in the extracellular space, the uptake can be categorized as autologous or heterologous (2018; Menck et al., 2015). Menck et al., showed that tumor-derived EVs are crucial for the invasion of tumor cells via both autologous- and heterologous-dependent communication (Menck et al., 2015). Although much research has focused on EV biogenesis, it is quite fascinating and well-understood how EVs are selected and taken up by recipient cells.

## APPLICATION OF EVs AS A DIAGNOSTIC AND THERAPEUTIC TOOL

The ability of EVs to carry endogenous proteins and nucleic acids and conduct their biological functions in recipient cells makes EVs an ideal candidate for diagnosis, therapeutics, and drug delivery.

### EVs as a diagnostic tool

Several reports indicate that EVs represent the content of their parent cells, and thus serve as a fingerprint or image of their originating cells (Salomon et al., 2015; Sharma et al., 2017). Based on this feature, EVs have been widely used as a diagnostic tool for various diseases, including glioma (Basu and Ghosh 2019). Glioma is a type of brain cancer that occurs in the CNS (Thakur et al., 2018) although the presence of the blood-brain barrier (BBB) limits the diagnosis and prognosis of glioma. With the benefits of their small size and ability to the cross the BBB, EVs are an important tool for early detection of glioma. As noted previously, several research groups isolated EVs from various biofluids, and blood serum- or CSF-derived EVs have shown promising results in tracking the malignant progression of parent glioma cells (Whitehead et al., 2017; Thakur 2019). Similarly, the constituents of EVs have been used as a diagnostic tool to track the progression of other types of cancer (Soung et al., 2017), such as exosomal-NY-ESO-1 in lung cancer (Sandfeld-Paulsen et al., 2016), exosomal-PKG1, Ral GTPase activating protein catalytic subunit alpha 2 (RALGAPA2), nuclear transcription factor, X-box binding 1 (NFX1), tight junction protein 2 (TJP2), human epidermal growth factor receptor 2 (Her2), Glypican-1 in breast cancer (Melo et al., 2015; Chen et al., 2017a; Fang et al., 2017), exosomal-Glypican-1 in pancreatic cancer (Melo et al., 2015), exosomal-Glypican-1, carcinoembryonic antigen (CEA) in colorectal cancer (Li et al., 2017; Yokoyama et al., 2017), exosomal-prostate specific antigen (PSA), glycoprotein galactosyltransferase alpha (GGTA) in prostate cancer (Kawakami et al., 2017; Logozzi et al., 2017), and exosomal-CD24, claudin-4, epithelial cell adhesion molecule (EpCAM), cancer antigen 125 (CA-125) in ovarian cancer (Li et al., 2009; Zhao et al., 2016). Other research has demonstrated that exosomal monocarboxylate transporter 1 (MCT1) and CD147 can be used as biomarkers to track metabolic reprogramming and progression of malignant glioma. The authors utilized different methods including localized surface plasmon resonance (LSPR) and atomic force microscopy (AFM) to detect the presence of MCT1 and CD147 in exosomes isolated from normoxic and hypoxic glioma cells. The study revealed the correlation between the level of MCT1 or CD147 in exosomes, which represented the gain or loss of function of MCT1 or CD147 in glioma cells, and the malignant phenotypes of glioma cells, such as migration and proliferation (Thakur et al., 2020c). Use of EVs as a diagnostic indication has gained much attention because they can be detected in liquid biopsy samples such as blood, urine, and CSF (Pang et al., 2020). Furthermore, their biophysical properties have been utilized for development of an additional diagnostic biomarker (Paolini et al., 2018), as seen in the change of roughness, stiffness, and adhesion force of EVs in hypoxic vs. normoxic conditions. Interestingly, the utilization of EV-based non-invasive liquid biopsy as a diagnostic method has recently gained much attention due to the presence of EVs in various biofluids including malignant glioma (Saenz-Antoñanzas et al., 2019).

### EVs as a therapeutic tool

The property of EVs to carry protein and nucleic acids that biologically affect recipient cells makes EVs an attractive therapeutic agent. EVs derived from some cell sources including mesenchymal stromal cells, DCs, and iPSCs, have shown therapeutic activities (Liu et al., 2017b; Yamashita et al., 2018). For example, mesenchymal stromal cell-derived EVs carry and deliver miRNAs or proteins such as interleukin-10 (IL-10) and TGF-β, resulting in anti-inflammatory effects by inhibiting cytokine expression, and tissue regeneration via increasing extracellular matrix modelling. Therefore, mesenchymal stromal cell-derived EVs hold therapeutic potential for several diseases such as cancer (Teng et al., 2015; Wang et al., 2017). In addition, they are relatively safer as they have a lower risk of forming teratoma and embolization (Sun et al., 2016; Liu et al., 2017b).

Several reports have explored the application of EVs as a vaccine. DC-derived EVs can be used as a vaccine against cancer, owing to their character as antigen-presenting cells (APCs). In the main, DC-derived EVs express major histocompatibility complex (MHC)-I, MHC-II, and co-stimulating factor (such as CD86; Théry et al., 1999, 2001). EVs isolated from mature DCs can induce a high T-cell response compared with those isolated from immature DCs (Segura et al., 2005). In addition, EVs isolated from DCs can activate natural killer cells via NKG2D ligands and IL-15Rɑlpha (Viaud et al., 2009). André et al. showed that DCs release profound MHC class I/peptide complexes in EVs to other naive DCs for adequate priming of CD8^+^ T cells *in vitro*. Moreover, mature DCs are required by EVs to facilitate the differentiation of melanoma-specific effector T lymphocytes, which could produce Interferon gamma (IFN-γ; Tc1) effector lymphocytes in human leukocyte antigen A2 (HLA-A2) transgenic mice (HHD2). Therefore, EVs could be associated with the transfer mechanism of functional MHC class I/peptide complexes to DCs for effective activation of cytotoxic T lymphocytes (CTL) *in vivo* (André et al., 2004).

Blocking pathological intercellular communication via EVs also holds therapeutic value. For example, an enhanced intracellular calcium level augments the release of EVs from K562 leukemia cells (Savina et al., 2003). Notably, blocking of H^+^/NA^+^ and Na^+^/Ca^+^ channels using dimethyl amiloride (DMA) dramatically reduces release of EVs in a mouse model of colon carcinoma, because these two channels control intracellular calcium levels (Chalmin et al., 2010). This suggests that developing techniques or drugs to obstruct the release of EVs has remarkable potential to inhibit cancer progression. Another study revealed that the enhanced level of MCT1 and CD147 in glioma cells could increase the release of EVs in an intracellular calcium-dependent manner. The enhanced level of MCT1 and CD147 in glioma cells is involved in the progression of glioma and their knock down reduces the migration and proliferation of glioma cells. Glioma cells exposed to hypoxia or lactate release a tremendously high level of EVs due to an enhanced intracellular calcium level. This was blocked by treating glioma cells with BAPTA-AM (a calcium channel blocker) as well as via genetic knockdown of MCT1 or CD147. This shows that targeting MCT1 and CD147 in glioma cells to reduce the release of EVs could be a crucial therapeutic target to inhibit glioma progression (Thakur et al., 2020c). Nonetheless in order to use EVs therapeutically, their large-scale production needs to be improved, as well as the ability to isolate EVs with high purity as well as yield, and optimization of storage conditions (Yamashita et al., 2018).

### EVs as a drug delivery carrier

Numerous reports have shown that EVs can be an efficient drug delivery vehicle, delivering various cargoes to the target cells. These EVs can be either naturally obtained or synthesized with modification to enhance their drug delivery efficiency (Bunggulawa et al., 2018), and enable delivery of active pharmaceutical ingredients or siRNA (Aryani and Denecke, 2016). Interestingly, the hydrophilic core of EVs is an optimum environment for loading hydrophilic drugs (Jiang and Gao, 2017). Owing to their homing property, EVs can deliver the cargoes to distant target sites.

The EV-based delivery system has several advantages in terms of safety, specificity, and stability. EVs can evade phagocytosis and destruction by lysosomes and induce minimal immune response, owing to their small size and native formation (Ha et al., 2016). In general, stability is an important parameter in the evaluation of any delivery system, especially in the human circulation; the EV-based delivery system is stable in blood, facilitating long-distance transportation (Jiang and Gao, 2017). Osteikoetxea et al. showed that among three different types of EVs, exosomes were the least sensitive to detergents (Osteikoetxea et al., 2015), suggesting their suitability as a delivery carrier. Loading of cargoes in EVs can be before or after isolation from donor cells (van der Meel et al., 2014), and various methods have been used such as passive loading methods (incubation of cargoes with EVs, or donor cells) and active loading methods (sonication, extrusion, freeze-thaw, electroporation, incubation with membrane permeabilizing agent, click chemistry, antibody-mediated conjugation; Luan et al., 2017). Thakur A et al. showed that anti-cancer drugs such as doxorubicin and paclitaxel could be loaded into SF7761 glioma stem-like cells in the presence of saponin (acting as a permeabilizing agent) via a microfluidic Exo-Load device (Thakur et al., 2018, 2020d).

Surface modification of EVs has also been carried out to accomplish various goals such as enhancing target capacity (Tian et al., 2018) and site-specific delivery (Nakase and Futaki, 2015). EVs combined with pH-sensitive fusogenic peptide and cationic lipid have been explored for cytosolic delivery (Nakase and Futaki, 2015). EVs have been engineered with polyethylene glycol (PEG) and aminoethylanisamide (AA) to target malignant pulmonary cancer, such as lung adenocarcinoma (Kim et al., 2018; Liu et al., 2018). Fusion of EVs with liposome increased therapeutic outcome due to the enhanced targeting ability of modified EVs (Sato et al., 2016; Dimov et al., 2017).

## ROLE OF EVs IN CANCER PROGRESSION

Cancer is the second leading cause of death in the United States, affecting approximately 1.7 million people with more than 600,000 associated deaths every year (Ma and Yu, 2006). Although the combination of chemotherapy, radiotherapy, and surgery is vital for cancer treatment (Ringborg et al., 2003), it is not effective in many cases (Vasan et al., 2019). The hallmarks of cancer include indefinite proliferation, evasion of apoptosis, angiogenesis, invasion, and metastasis (Chaffer and Weinberg, 2011; Baskar et al., 2012). One of the major reasons for treatment failure and cancer-related death is metastasis (Steeg, 2016). Cancer metastasis entails a series of stages controlled by cellular or molecular events. These include remodeling of the ECM, epithelial-mesenchymal transition (EMT), immune response, reprogramming of the tumor microenvironment, and involvement of bone marrow-derived cells such as MSCs in some cancers (Peinado et al., 2017). Notably, cancer cells employ angiogenesis, the growth of blood vessels from existing vasculature, to acquire nutrition and eliminate waste products from the tumor, facilitate survival and proliferation of cancer cells, and connect to the whole body circulation for distance trafficking and metastasis (Tonini et al., 2003).

Cancer cell-derived EVs and communication play an important role (He et al., 2019) in angiogenesis, migration, invasion, proliferation (Tian et al., 2019), immunomodulation (Baquir and Hancock 2017; Othman et al., 2019), and drug resistance (Zhang et al., 2018) in cancers. Mao et al. showed that EVs isolated from hypoxic esophageal squamous cell carcinoma (ESCC) cells promoted metastatic phenotypes by increasing tube formation (a measure of angiogenesis) of human umbilical vein endothelial cells (HUVECs; Mao et al., 2019). Ko et al. demonstrated that cancer cell (ovarian, colorectal, and renal cancer cell lines)-derived EVs could increase angiogenesis of endothelial cells (ECs) independent of EV uptake, indicating that EV uptake is not necessary to produce a functional change in the recipient cells (Ko et al., 2019). The phenotypic response was induced by a 189 amino acid-isomer of vascular endothelial growth factor (VEGF_189_), favorably localized on the membrane of EVs via its affinity to heparin. The interaction between VEGF_189_ and EVs significantly augmented the half-life of the ligand and decreased its recognition by bevacizumab (a VEGF antibody). Therefore, tumor angiogenesis stimulated by EV-associated VEGF (VEGF-EVs) could not be neutralized by bevacizumab. Moreover, the augmented VEGF-EVs were linked to lethality of disease when patients were treated with bevacizumab, suggesting that resistance to bevacizumab was potentially due to raised amounts of VEGF-EVs (Ko et al., 2019). Another study by Deng et al. showed that miR-155 in exosomes isolated from gastric carcinoma cells caused angiogenesis via targeting of the c-MTB-VEGF pathway in ECs, indicating that miR-155 in EVs can be a potential therapeutic target for gastric carcinoma (Deng et al., 2020). In addition, recent research has demonstrated the functionality of ovarian cancer-derived EVs carrying miR-205 that induce angiogenesis via regulation of the PTEN-AKT pathway in ECs *in vitro* and *in vivo* (He et al., 2019). Conclusively, the cancer-derived EVs can promote angiogenesis by carrying various functional cargoes and delivering them to recipient cells. Therefore, potential approaches for cancer treatment should target the release of EVs from donor cells, the pathways they regulate, or their uptake by recipient cells.

Several studies have suggested the role of remnant CSCs in the relapse or recurrence of cancer, tumor growth, metastasis, and drug resistance (Chang 2016), including the role of EVs in the progression of cancer (Wu et al., 2017). Certain stem cells including MSCs release EVs that facilitates communication in the tumor niche and plays a diverse role in metastasis, angiogenesis, and tumor formation. Nonetheless other studies have described the tumor-suppressing capacity of EVs derived from MSCs (Vakhshiteh et al., 2019). Therefore, the role of MSC-derived EVs is dependent on cell context and tissue microenvironment. It is important to understand the exact crosstalk among niche cells in the tumor microenvironment and the functional constituents of EVs during cancer progression. Several studies have suggested a potential role of EVs in assisting the formation of a pre-metastatic niche in the tumor microenvironment during cancer metastasis (Peinado et al., 2012; Costa-Silva et al., 2015; Steeg, 2016; Anderson et al., 2019). Costa-Silva et al., demonstrated that EVs from pancreatic ductal adenocarcinoma (PDAC) could initiate the generation of a pre-metastatic niche in the liver of naive mice and thereupon enhance the liver metastatic burden. The PDAC-derived EVs were engulfed by Kupffer cells causing secretion of TGF-β and enhancing the production of fibronectin by hepatic stellate cells. The fibrotic microenvironment promoted the use of macrophages derived from bone marrow. Interestingly, the PDAC-derived EVs contained a high level of macrophage migration inhibitory factor (MIF), and its impediment could obstruct the formation of a liver pre-metastatic niche (Costa-Silva et al., 2015).

## TECHNOLOGICAL ADVANCEMENTS IN EV RESEARCH

Recent advances in technology have made it possible to effectively and efficiently isolate and analyze EVs (Ludwig et al., 2019). In general, the exosomes are isolated from cell culture conditioned medium (CCM) or biofluids using differential ultra-centrifugation, density gradient zone centrifugation, immunocapture by magnetic beads, exosome precipitation, or chromatography (Witwer et al., 2013; Yu et al., 2018).

### Differential ultra-centrifugation

Differential ultra-centrifugation is a simple and widely used method that involves the centrifugation of CCM at different speeds to remove cell debris and obtain exosome pellets (Théry et al., 2006). Despite its widespread use, it is labor-intensive, tedious, reliant on a heavy centrifuge machine, and reduces yield because of damage to exosomes due to repeated centrifugation (Merchant et al., 2017). The method is not suitable for the isolation of exosomes from a small volume sample such as biofluids (blood serum; Witwer et al., 2013) or CSF. Another major hurdle of this method is the purification of exosomes from MVs or ABs, as the size of exosomes overlaps that of MVs and ABs (Zhang et al., 2017). There is a continuous need for better alternatives to isolate different types of EVs.

### Density gradient zone centrifugation

Density gradient zone centrifugation is another means to isolate exosomes where the sample is subjected to centrifugal sedimentation after adding an inert gradient-based medium. Sucrose-based density gradient zone centrifugation is the most commonly employed method, where different components of the samples, including exosomes, are separated based on their density in the various iso-density zones (Kamerkar et al., 2017; Momen-Heravi, 2017). Compared with differential ultracentrifugation, this method separates exosomes with better purity and higher efficiency. Additionally, the separated components will not re-mix and the probability of damage to the exosomes is negligible. Nonetheless the method has limitations. The process is time-consuming, the yield of exosomes is relatively low, the intricate process requires preparation of the inert gradient medium in advance, and instruments required to perform the experiments are costly. These factors prohibit widespread use of this method (Zhang et al., 2017).

### Precipitation method

Precipitation method is another widely employed method for isolating EVs. It is convenient and straightforward with various kits available commercially, such as Exo-Quick (System Bioscience), and Total Exosome Isolation reagents (Thermo Scientific). The essential process requires mixing samples with reagents followed by simple centrifugation steps. The precipitation method is useful for isolating exosomes from low volume samples. Nonetheless the reagents for exosome isolation become expensive if the process is scaled-up for a large sample size (Safdar et al., 2016).

### Size exclusion chromatography

Size exclusion chromatography has also been used to isolate exosomes in various biofluids. The large particles, including cellular debris, are removed by centrifugation at low speed, followed by filtration with filters of pore size 0.8 and 0.2 µm. The resultant pellets of EVs are yielded by running through a gel filtration column, followed by ultracentrifugation at around 100,000 ×*g* for one hour. Despite its prevalent use, the method has several disadvantages. For example, EVs may be damaged during the filtration step. In addition, the method requires the selection of a suitable gel to exclude contaminants during isolation of EVs, and an ultracentrifugation machine is heavy (Böing et al., 2014; de Menezes-Neto et al., 2015; Lozano-Ramos et al., 2015; Szatanek et al., 2015).

### Immunocapture based isolation of EVs

Immunocapture based isolation of EVs has also gained large attention mainly because of its ability to maintain the integrity of exosomes, as no repeated centrifugation steps are involved. In brief, immunomagnetic beads are coated with a primary antibody that binds to the specific target proteins on the surface of EVs, followed by incubation with the samples. Subsequently, a magnetic field is applied to separate the captured exosomes on the surface of immunomagnetic beads. In general, exosomal marker proteins such as CD9, CD63, and CD81 are utilized to isolate exosomes. This method consumes relatively less time and enables isolation of a specific population of exosomes based on their surface markers. Nonetheless the major disadvantage is the low elution yield of exosomes captured on immunomagnetic beads, making the downstream analysis a challenge.

Recently, efforts have been made to explore the possibility of combined approaches to isolate EVs to enhance their yield and reduce the time required. Benedikter et al. showed that the combination of ultrafiltration and size exclusion chromatography could efficiently isolate EVs from cell culture conditioned medium (Benedikter et al., 2017).

Several other advanced methods have been developed to isolate exosomes, such as integrated microfluidic device (Liang et al., 2017b), nanoplasmon-enhanced scattering (nPES; Liang et al., 2017a), membrane-mediated exosome isolation (Zhang et al., 2017), and on-chip exosome isolation (Liu et al., 2017a). Liang L-G et al. demonstrated a double-filtration device to isolate EVs from urine samples, detected by a microchip enzyme-linked immunosorbent assay (ELISA) method integrated with a cell phone. This integrated device could distinguish bladder cancer samples from healthy samples by integration with advanced sensing techniques, such as surface plasmon resonance (SPR; Liang et al., 2017b). Another study by Liang K et al. demonstrated a fast, ultrasensitive and low-cost nPES assay for direct quantification of cancer-derived EVs from plasma with a volume as low as around 1 μL. This method captured EVs using specific antibodies in a sensing chip, leading to the generation of a local plasmonic effect, thus increasing the sensitivity and specificity for detection of cancer-derived EVs. This technique was also reported to distinguish pancreatic cancer patients from healthy subjects via detection of an EV marker, ephrin type-A receptor 2 (EphA2), associated with pancreatic cancer for clinical diagnosis and prognosis (Liang et al., 2017a). Liu et al. demonstrated that a size-based EV isolation device, referred to as an exosome total isolation chip (ExoTIC), could isolate EVs with high-yield and high-purity from both cell lines and clinical biofluid samples including plasma, urine, and lavage. Importantly, this method was effective in isolating EVs from samples with small volume, and thus crucial for point-of-care diagnosis of cancer and other diseases (Liu et al., 2017a).

Besides isolation, advanced techniques have been developed to characterize the size and morphology, and identify the cargoes of EVs such as protein, long non-coding RNA (lncRNA), and micro-RNA (miRNA; Xu et al., 2016). The quantification of EVs depends on either the whole protein by using bicinchoninic acid (BCA)-, Bradford-, and detergent compatible protein- assay, or the number of EV particles analyzed by a nano-tracking analyzer (NTA; Koritzinsky et al., 2017). NTA can determine the size range as well as distribution of EVs although other techniques are available such as differential light scattering (DLS), transmission electron microscopy (TEM), flow cytometry, and resistive pulse sensing (RPS; van der Pol et al., 2014). The morphology of EVs can be determined using TEM, cryo-EM, and scanning electron microscopy (SEM; Noble et al., 2020). Other molecular techniques are available to identify constituents of EVs. Immunogold-EM is used for the qualitative detection of specific proteins, whereas Western blot and ELISA are used for quantification of specific proteins. The advantages and disadvantages of various methods used for characterization of EVs are listed in Table 1.

**Table 1:** Advantage and disadvantages of methods for characterization of EVs.

**Method**	**Characteristic of EVs**	**Advantages**	**Disadvantages**	**Ref.**
NTA	Particle size distribution; concentration (number of particles)	Detect particle in the size range of 10–1000 nm diameter	Requires sample volumes around 500 µL Requires optimization for collection of data and parameters of analysis	(Soo et al., 2012) (Palmieri et al., 2014) (Filipe et al., 2010)
DLS	Size distribution; zeta potential	Requires very small sample volume (70 µL) Easy to use (requires optimization for a few parameters)	Poor analysis of heterogeneous populations of particles	(Palmieri et al., 2014) (Filipe et al., 2010)
Tunable Resistive Pulse Sensing (tRPS)	Size distribution, concentration of particles	Length of the resistive pulse is correlated with the particle size Rate of resistive pulses reveals concentration of particles	An indirect method that requires a series of standard sample	(Maas et al., 2014)
TEM	Size, morphology	Produces high resolution images Electrons that pass through the sample are detected	EVs typically have a divot in their center due to the drying process associated with the sample preparation	(Wu et al., 2015)
SEM	Size, morphology	Produces high resolution images Scattered electrons are detected	Requires extensive sample preparation EVs typically have a divot in their center due to the drying process associated with the sample preparation	(Wu et al., 2015)
Cryo-EM	Size, morphology	Samples can be conserved in their native hydrated state Produces better quality and preserved morphology Artifacts can be avoided In combination with TEM, cryo-EM can detect proteins in EVs, and uptake by cells	Requires extensive sample preparation	(Chernyshev et al., 2015; Choi and Mun 2017; György et al., 2017)
Immunogold-EM	Specific protein detection qualitatively	Requires small volume of EVs Can detect the proteins in EVs Can detect multiple proteins in EVs by using different size secondary gold particles Quantify disease specific markers in EVs Better for molecular characterization of EVs	Requires extensive sample preparation	(Cappello et al., 2016)
Western blot	Specific protein detection quantitatively	Allows molecular characterization of EVs. Allows quantification of proteins in EVs.	Does not allow observation of intact vesicles Not well multiplexed The specificity and reproducibility are limited by the quality of the antibody used Requires large sample volume Extensive sample processing is required Specialized instruments are needed	(Gallagher et al., 2008)
ELISA	Specific protein detection quantitatively	Allows quantification of protein in EVs, crucial for molecular characterization of EVs	Requires a large sample volume Extensive sample processing is required Specialized instruments are needed	(Witwer et al., 2013)
Flow cytometry	Specific protein detection quantitatively	Detection limit is 100–200 nm Allows for high throughput analysis of exosomes Allows for quantification or classification of exosomes based on the antigen expression	Requires a single particle suspension Aggregation of vesicles results in the observation of multiple particles at a single time Requires the immobilization of exosomes on the surface of beads	(Szatanek et al., 2017) (Ko et al., 2016)
Thermophoretic aptasensor (TAS)	Profile EVs as a function of surface protein expression	Inexpensive, fast, and requires small serum volume (less than1 µL)	Currently, TAS profiles one marker per run. Therefore, further development is necessary for high throughput Accuracy needs to be further improved.	(Liu et al., 2019)
Mass spectroscopy	Proteomic analysis of EVs	Allows the identification and quantification of thousands of EV proteins Can identify missing proteins in the human protein map	Protein interference issue due to the identification of peptides as protein surrogate sequence coverage Requires isolation of homogeneous EV population Characterization of the proteome of EVs isolated from primary cell lines and tissues is challenging	(Rosa-Fernandes et al., 2017)
SPR	Membrane protein analysis, biophysical properties, protein-protein interaction	Real-time measurement Able to detect low affinity antibodies or antigens, a calibration-free concentration analysis Elimination of labels Requires low sample volume	Requires high sensitivity and specificity for the detection of biomarker at the early stage of disease progression The sensor chip requires functionalization of ligands	(Thakur et al., 2017; Hosseinkhani et al., 2017; Im et al., 2014)
AFM	Membrane protein analysis, biophysical properties, topology, surface characteristics	Can detect EVs in liquid as well as air mode Produces topographical pictures of EVs Allows quantification and imaging of EVs Specific EVs can be detected via immobilization of antibodies Extensive sample preparation is not required Resolution limit is around 1 nm	Requires specific stages such as mica for immobilization. Requires probe for the detection of EVs, which can damage the EVs	(Klinov and Magonov 2004; Sharma et al., 2010, 2011; Yuana et al., 2010; Hardij et al., 2013)
Raman spectroscopy	Detects membrane protein, functionality	Simple, inexpensive, highly efficient, and portable method	Analysis of a single vesicle is time-consuming because of the weak Raman signals that often need enhancement via the nanostructured substrates or nanoparticles for a more effective analysis	(Kwizera et al., 2018; Gualerzi et al., 2019)
Quantum dots	Detection of disease specific exosomes	Sensitive detection of 100 exosomes per μL Facilitates better tracking of EVs and more specific targeting QDs have strong resistance to photobleaching	In the context of QD-EV conjugation chemistry, the NHS-ester used for QDs and EV modification can react with primary amines.	(Boriachek et al., 2017; Goreham et al., 2020; Zhang et al., 2020) (Takov et al., 2017)
Integrated magneto-electrochemical sensor (i-MEX)	Fast and streamlined analysis of EVs Cell-specific exosomes can be isolated High detection sensitivity through magnetic enrichment and enzymatic amplification Sensors can be miniaturized	Fast, high-throughput, and on-the-spot analysis Cell-specific exosomes can be isolated directly from complex media High detection sensitivity via magnetic enrichment and enzymatic amplification Can be miniaturized and expanded for simultaneous measurements	The iMEX system has lower sensitivity and throughput than nPLEX	(Jeong et al., 2016)
Aptamer based biosensor	Quantitative detection of exosomes	Requires small sample volume Application of aptamer instead of antibody, improves the stability of the system, resulting in better sensitivity Due to label-free approach, the cost is reduced. The aptasensor detected exosomes in a homogeneous system	Lack of a reliable process to obtain aptamers to be specifically used in electrochemical sensors	(Zhou et al., 2016; Rozenblum et al., 2019; Xia et al., 2017)
Aptasensor	Detects exosomes by integrating single-walled carbon nanotubes	Visible and simple method Can be applied to detect other targets by changing the aptamer	Requires development of a “signal-on” strategy to replace “signal-off” strategy, susceptible to interference	(Xia et al., 2017)

Moreover, some advanced tools, such as SPR and AFM, have been applied to analyze EV-proteins as well as their biophysical properties, such as adhesion force, stiffness, and roughness (Sharma et al., 2014). SPR allows real-time, label-free recognition of proteins (on the surface of EVs) binding to target antibodies that are immobilized on the surface of the sensing chip (Im et al., 2017). AFM has been widely employed to characterize morphology, biomolecular constituents, and biomechanics of EVs, because of its prolific role in the study of biomarkers and therapeutics (Sharma et al., 2018). In addition, Raman spectroscopy has been used to study EVs and is based on measurement of the scattered photons by vibration after exposing samples to a monochromatic laser light. The photons alter their energy by excitation of molecular vibration, resulting in a Raman spectrum that enables determination of the molecular composition of a sample by comparing results with known vibration modes of specific chemical groups. Importantly, this method is label-free (without the need to tag the molecule of interest), does not distort the morphology of EVs, and enables analysis of exosomes in the context of the exosomal membrane lipid/protein content along with other modifications on the surface (Carmicheal et al., 2019).

Artificial intelligence (AI) has recently been applied in EV research (Thakur et al., 2020a, 2020b) for clinical diagnosis and prognosis, as disease specific parent cells can be identified using an AI-based classification approach and datasets of EVs (Thakur et al., 2020a).

## MICROFLUIDIC SYSTEMS IN EV RESEARCH

EVs occupy a crucial position amidst the biomarker studies due to their clinical utility for the diagnosis and prognosis of diseases (Lane et al., 2018; Ma et al., 2019). Nonetheless, there is a dearth of robust systems by which to achieve their convenient and efficient isolation, characterization, and examination.

A promising technique to solve this problem is the microfluidic system. A microfluidic system is a device with separation and sensing features. It can perform isolation, detection, and analysis of EVs in one single platform. In particular, the microfluidic technique has a broad range of applications in the development of point-of-care devices for clinical use, and offers a new avenue to use EV-based liquid biopsy for personalized medicine (Contreras-Naranjo et al., 2017). Typically, the microfluidic-based platforms contain various components for isolation of EVs, such as membrane-based filtration, acoustic nano-filtration, immunoaffinity, deterministic lateral displacement (DLD) sorting, and trapping on nanowires (Contreras-Naranjo et al., 2017). In the membrane-based filtration microfluidic system, many techniques have been developed to better facilitate the isolation of EVs, such as pressure-driven filtration, electrophoresis-driven filtration (Davies et al., 2012), electrophoretic isolation on the nano-porous membrane (Cho et al., 2016), and double filtration (Liang et al., 2017b; Woo et al., 2017). Lee, Kyungheon et al. (2015) utilized differential ultrasound forces in an acoustic nano-filter system that was integrated into a microfluidic device to isolate EVs by size in a contact-free manner (Lee et al., 2015). Further, various microfluidic systems have been designed for immunocapture of EVs by targeting their specific surface markers (Kanwar et al., 2014). For example, the antibodies against CD24, CD63 or EpCAM, conjugated on the gold surface with nanohole arrays (reusable nPLEX), can facilitate capture of specific populations of EVs (Im et al., 2014). The capture of anti-CD9, anti-HER2 antibodies on the functionalized gold electrodes could be enhanced by a nano-shearing approach (Vaidyanathan et al., 2014). A study by Mei et al. reported isolation of EVs using antibodies targeting other EV surface proteins, such as insulin like growth factor 1 receptor (IGF-1R), CA125, CD9, CD63, and CD81, using immunomagnetic microbead (He et al., 2014). A microfluidic device was developed to specifically isolate a subpopulation of exosomes from plasma samples and quantitatively detect surface biomarkers with improved sensitivity (He et al., 2014). The application of rapid inertial solution exchange (RInSE) was also employed for the isolation of EVs via EpCAM based affinity-capture microbeads (Dudani et al., 2015). For DLD sorting in the microfluidic system, an array of pillars has been utilized to isolate EVs (Wunsch et al., 2016). For instance, ciliated micropillars were employed in a microfluidic system to trap EVs on nanowires (Wang et al., 2013). Overall, various miniaturization- and functionalization- based EV isolation approaches have augmented yields, consumed less time, and attained better purity.

The integration of the microfluidic technique with other methods has also improved the analysis of EVs. For example, the sequential ExoChip that combines immunoaffinity-based isolation of EVs and fluorescent dye-based staining can isolate and detect EVs in one process (Kanwar et al., 2014). Another study demonstrated the integration of immunoaffinity-based EV isolation and fluorescence immunoassay in sequential nano-interfaced microfluidic exosome (nano-IMEX; Zhang et al., 2016a). Similarly, better analyses of EVs was achieved by a sequential Exodisc that combined double filtration isolation and colorimetric ELISA (Woo et al., 2017). In addition, an integrated microfluidic system for the analysis of miRNA in EVs was developed and combined sequential iMER, immunomagnetic isolation, exosome lysis, RNA capture, and multiplexed RT-qPCR. Advancements in the development of microfluidic systems have solved various issues pertaining to EV-based studies. Nonetheless several challenges remain to be addressed before it can be used in routine clinical practice such as antibody free processing, integration of microfluidic system with downstream analysis, standardization of techniques, and the possibility of commercialization (Contreras-Naranjo et al., 2017).

## CONCLUSION AND FUTURE PERSPECTIVES

EVs play important roles in stem cell biology, including intercellular communication in the stem cell-niche and at various stages of embryonic development. In addition, the role of EVs in cancer progression has begun to be elucidated. Studies of the application of EVs as a diagnostic and therapeutic tool have gained attention due to their numerous outstanding advantages. For example, EVs can be isolated from body fluids (such as blood, CSF, and urine) non-invasively, and continuous time-dependent monitoring of a patient’s response is possible with EV-based therapeutics. Because of their small size, EVs can cross physiological barriers, including the BBB, making them useful in drug delivery. The benefits of EVs in therapy of various diseases including cancer have also been studied. Other critical aspects of the application of EVs involve their self-renewal capacity, ability to arbitrate communication between stem cells and niche, and their functions in stem cell differentiation or cell fate determination. The role of EVs in normal physiological processes and disease conditions has also been extensively studied, including malignant progression of cancer. The exploration of various multidimensional roles of EVs have become possible with technological advancements in methods for isolation and characterization of EVs, and the development of advanced microfluid based systems for studies associated with EVs.

The role of EVs has yet to be thoroughly explored. In the study of disease progression via intercellular communication of EVs, the potential of EVs to carry various cargoes to recipient cells warrants attention. In the context of cancer progression, there are several drugs such as paclitaxel, which causes neuropathic pain, or drugs against which drug resistance has developed such as temozolomide for glioma, that could be a cargo for EVs. Furthermore, the exploration of potential changes in the molecular constituents of stem cells, after uptake of EVs released from a different subset of cancer cells, may help future research into the mechanisms that underlie the development of drug resistance.

The use of EVs as a carrier for either genetic materials such as lncRNA, miRNA, or small molecules such as anti-cancer drugs, holds great promise for the development of novel delivery methods. One of the important advantages of EVs as a delivery carrier is their ability to escape the reticuloendothelial system (RES). Nonetheless the synthetic nano-delivery systems are often prone to be captured by RES and therefore very likely to prompt an immune response. In the prevailing scenario, the use of EVs, particularly autologous EVs, has strong potential for targeted delivery after surface modifications with specific ligands against the target receptor, for example, epidermal growth factor receptor (EGFR) in lung cancer and EGFRvIII in glioma.

The application of EVs to track disease progression, including metastatic progression of cancer, is an exciting prospect. The conventional approaches to diagnosis of cancer, such as investigation of molecular changes in tissues via biopsy, imaging techniques like magnetic resonance imaging or computed tomography in glioma, and digital rectal examination in prostate cancer, are limited by their resolution in detecting the development of tumor. In the prevailing scenario, the development of biosensors is required to detect EVs and their constituents with high sensitivity and specificity for tracking disease diagnosis. Additionally, mapping of various proteins on the surface of EVs could be used to potentially demarcate the disease state.

Although there is significant ongoing research associated with EVs, there remains a need to develop a universal protocol for their isolation and characterization, as well as for their clinical application. When available, it could be certified and applicable for good clinical practice (GMP). A specific road map could be developed for the translation of EVs from bench to bedside.

## Abbreviations

EPCs: endothelial progenitor
ESCs: embryonic stem cells
EVs: extracellular vesicles
HSCs: hematopoietic stem cells
MSCs: mesenchymal stem cells
MVBs: multivesicular bodies
NSCs: neural stem cells
SSCs: somatic stem cells
